# Template-free synthesis and lithium-ion storage performance of multiple ZnO nanoparticles encapsulated in hollow amorphous carbon shells†

**DOI:** 10.1039/d0ra02497j

**Published:** 2020-06-15

**Authors:** Yunxia Jin, Shimin Wang, Jia Li, Sheng Qu, Liufang Yang, Junming Guo

**Affiliations:** School of Electrical and Information Technology, Yunnan Minzu University Kunming 650500 China jyx1601@aliyun.com; School of Chemistry and Environment, Yunnan Minzu University Kunming 650500 China guojunmingkm@163.com; National and Local Joint Engineering Research Center for Green Preparation Technology of Biobased Materials, Yunnan Minzu University Kunming 650500 China

## Abstract

Due to the limited utilization of electrode materials, the rational design and facile synthesis of composite structures are still challenging issues for lithium-ion batteries (LIBs). Herein, a simple approach has been developed to prepare multiple core–shell structures of ZnO nanoparticles (NPs) encapsulated in hollow amorphous carbon (AC) shells. The as-synthesized ZnO@AC composites showed a uniform dispersion of ZnO NPs, compliant buffer AC shells, and nanoscale void spaces between the ZnO NP cores and AC shells. As a result of their structural merits, the ZnO@AC composites were evaluated as anode materials for LIBs and delivered enhanced coulombic efficiency, high reversible capacity, high rate capability, and improved cycling stability.

## Abstract 2

Core–shell structure of ZnO@amorphous carbon shell was synthesized using a simple and effective method, and exhibited excellent electrochemical performance as anode of lithium-ion batteries.

## Graphical abstract

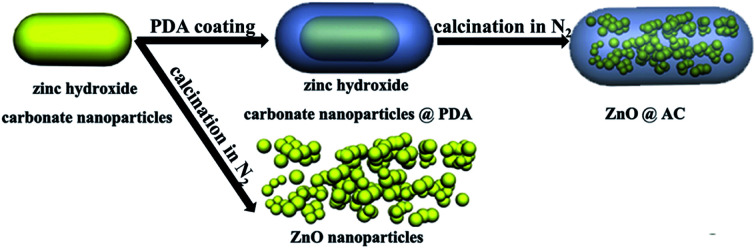

## 1. Introduction

Owing to the increasing demand for high-efficiency and renewable lithium-ion batteries (LIBs), the lithium storage technology has become a constant global concern over the past decades.^1,2^ A variety of inorganic materials have been developed to improve the stability and energy density of electrodes for LIBs.^3–5^ The hybrid multi-core–shell nanostructure has become one of the most promising candidates for LIBs due to its variable composition, large surface area, the controllable size of the inner nanomaterial, functional shell and the nanoscale void spaces in the shell.^6,7^ Therefore, considerable efforts have been devoted to exploring various multi-core–shell composites as electrode materials for advanced energy storage and conversion applications in LIBs. Recently, multi-core–shell nanostructures composed of transition metal oxides, such as SnO_2_,^8^ Fe_2_O_3_,^9^ Fe_3_O_4_,^10^ Co_3_O_4_,^11^ V_2_O_5_,^12^ and MoO_2_,^13^ have been investigated as the anode materials in LIBs. This evidences that distinguished fabrications are responsible for the superior LIB performances of capacity retention, rate performance, and coulombic efficiency. Among the numerous alternatives to traditional or commercial anodes, ZnO nanostructures exhibit many opportunities and possibilities for charge storage due to their high theoretical capacity (987 mA h g^−1^), relatively low cost, and natural abundance.^14–19^ Even so, similar to other metal oxides, ZnO has inherently poor electrical conductivity and undergoes undesirably large volume expansion upon cycling, which limit its energy storage performance.^20^ The fabrication of ZnO-based composite electrodes has been proved to be an effective way to improve energy storage performances,^21,22^ but significant challenges remain. Many of the intensive approaches employed to date have been focused on the preparation of ZnO/carbon composite electrodes using MOF-derived approaches,^23–26^ nanoparticle assembly,^27^ atomic layer deposition,^28^ chemical solution reactions,^29,30^ solvothermal methods,^31^ and electrostatic assembly.^32^ In these studies, carbon shells have been reported to effectively improve the Li ion storage capability by accelerating the electron transfer, accommodating the volume change, and controlling the solid electrolyte interphase (SEI) formation. Accordingly, the above-mentioned reports on ZnO/carbon composites have inspired the exploration of the rational design and delicate assembly of composites with homogeneously dispersed ZnO on inactive carbon-based matrices or layers. Moreover, the quest for new methodologies to synthesize ZnO-based composites continues to be great impetus to research efforts.

In this work, we have reported a novel and facile strategy to synthesize multiple ZnO nanoparticles encapsulated in hollow amorphous carbon (ZnO@AC) nanocomposites through two steps: (1) dopamine self-polymerization to form adherent polydopamine (PDA) shells on the surface of nanoscale zinc hydroxide carbonate precursors in an aqueous solution and (2) the carbonization of PDA accompanied by the decomposition of zinc hydroxide carbonate by annealing under N_2_. In the resultant structure, ZnO nanoparticles as the active materials were homogeneously embedded in hollow amorphous carbon shells. The introduction of the amorphous carbon shells improved the electrical conductivity of the composites, protected the inner active materials, and allowed the easy diffusion of Li ions. Furthermore, the void spaces between ZnO NPs and AC shells could offer compliant buffer for volume variation upon Li-ion insertion–extraction. The above-mentioned structural merits ensured improved energy storage performance. As a consequence, the ZnO@AC composites exhibited high reversible capacity, excellent rate capability, and stable cycling stability.

## 2. Experimental section

### 2.1 Sample preparation

All reagents were of analytical grade, purchased from Shanghai Aladdin Co. Ltd., and used without further purification. In a typical synthesis process, an oxalic acid solution (50 ml, 0.15 M) was added to a zinc acetate dihydrate solution (50 ml, 0.15 M) drop by drop under stirring. The mixed solution was stirred at room temperature for 8 h. The obtained white precipitate was centrifuged, washed several times and subsequently dried at 60 °C for 12 h in an oven, and zinc hydroxide carbonate nanoparticles were formed (Fig. S1 and S2†). The formed zinc hydroxide carbonate nanoparticles (1 g) were dispersed in DI water (50 ml) under ultrasonic treatment for 30 min. Tris-buffer (50 ml, 10 mM, pH 8.5) and dopamine (1 g) were successively added to the above suspension, which was stirred at room temperature for 24 h. Then, polydopamine (PDA) was coated onto the surface of the zinc hydroxide carbonate nanoparticles. Afterwards, the products were centrifuged and washed with deionized water several times and then dried at 60 °C for 12 h. Finally, the ZnO@AC composites were obtained by annealing the as-synthesized products at 700 °C for 3 h in N_2_. ZnO NPs used for the measurements were obtained by annealing the as-synthesized zinc hydroxide carbonate nanoparticles at 700 °C for 3 h in N_2_.

### 2.2 Material characterization

The morphology of the samples was characterized by transmission electron microscopy (TEM, FEI Tecnai G2 F30 Twin). TEM samples were prepared by dispersing the samples in ethanol; then, they were dipped in formvar stabilized with carbon support films. The loading amounts of carbon on the composites were examined by thermogravimetry (TG) analysis using a thermal analyzer (Netzsch TA449F3) instrument at a heating rate of 10 °C min^−1^ in air. The temperature range was 30–900 °C. X-ray powder diffraction (XRD) patterns were recorded using a diffractometer (Bruker D8 Advance A25X) equipped with a Cu Kα radiation source (*λ* = 1.5406 Å). The Brunauer–Emmett–Teller (BET) surface areas and pore size distributions were measured by nitrogen adsorption and desorption using a Belsorp-max instrument (BEL Japan Inc.,). Raman spectra were measured using a Renishaw inVia-Reflex instrument with a laser excitation wavelength of 532 nm.

### 2.3 Electrochemical measurements

The lithium ion storage properties were investigated by assembling 2032 coin cells. The working electrode was prepared by mixing the active material (70 wt%), acetylene black (20 wt%), and polyvinyl difluoride (PVDF, 10 wt%) in a grinder to form a slurry. The slurry was coated on copper foils and dried at 70 °C for 12 h and then punched into circular electrodes with the diameter of 12 mm. The loading mass of the electrode film was about 1.2 mg cm^−2^. The cells were assembled in a glovebox filled with Ar using lithium metal as the counter and reference electrodes, the Celgard 2300 membrane as the separator, and 1.0 M LiPF_6_ in ethylene carbonate (EC) and dimethyl carbonate (DMC) mixed solvent (1 : 1 in weight) as the electrolyte. The cycling and rate performances were recorded on a Land battery measurement system (Wuhan, China) with a cut-off voltage of 0.001–3 V *vs.* Li^+^/Li, and the capacities were calculated based on the total mass of the composites. Cyclic voltammetry (CV) curves were recorded on an electrochemical workstation (CHI 660D) at a scan rate of 0.2 mV s^−1^ between 0.001 and 3 V. All electrochemical experiments were performed at a constant temperature of 25 °C.

## 3. Results and discussion

**Fig. 1:**
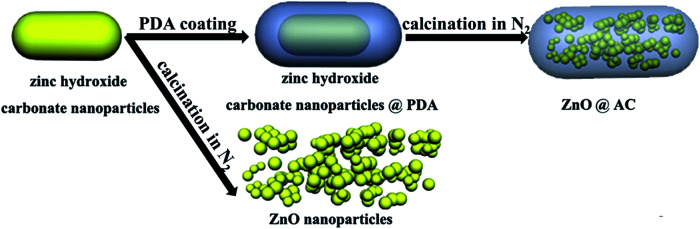
Schematic of the fabrication process of ZnO@AC and pure ZnO NPs.

**Fig. 2:**
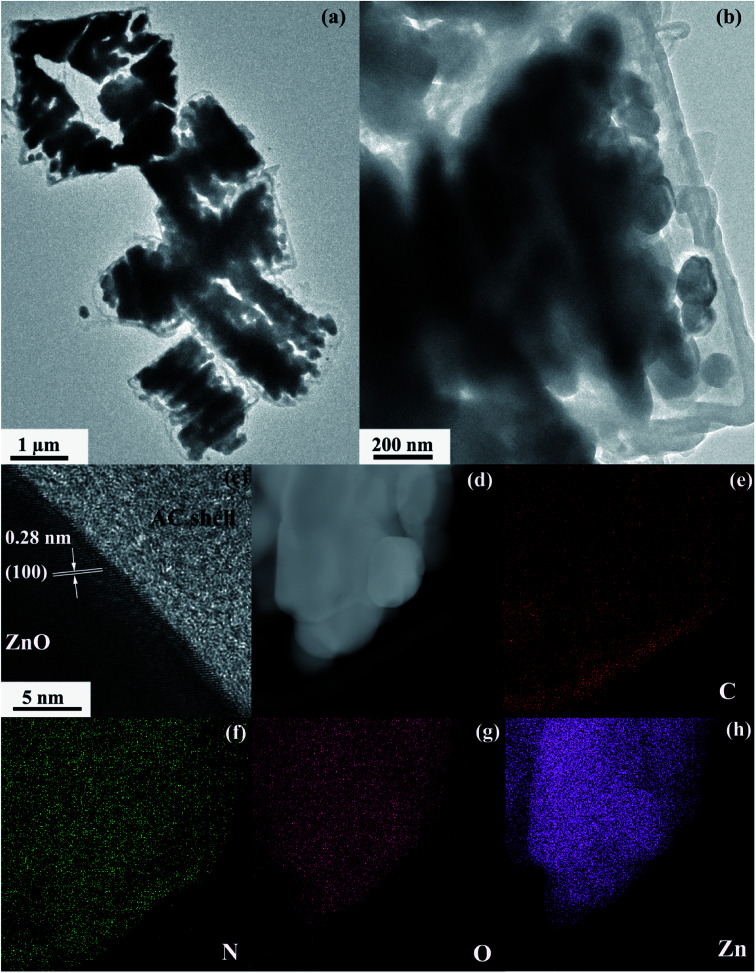
(a) Low-magnification and (b) high-magnification TEM images of the ZnO@AC composites. (c) HRTEM image of the lattice structure of the ZnO nanocrystal. (d–h) HAADF-STEM image and the corresponding C, N, O, and Zn elemental mapping of ZnO@AC.

A schematic of the formation procedures of the ZnO@AC composites and pure ZnO NPs is shown in Fig. 1. The ZnO@AC composites were synthesized using the sol–gel process of dopamine polymerization on the surface of zinc hydroxide carbonate nanoparticles, the subsequent carbonization of PDA and the thermal decomposition of zinc hydroxide carbonate in the same process of annealing under N_2_. In the annealing process, the zinc hydroxide carbonate nanoparticles decomposed and formed ZnO NPs with huge weight loss, which led to significant volume shrinkage and created complex void spaces inside the shells. During this thermal decomposition process, mixed gases of CO_2_ and H_2_O were released and passed through the PDA shells. As a consequence, multiple pores and channels were formed on the shells, which could be proved by the BET results. In the following heating procedure, the porous PDA shells were carbonized and formed amorphous carbon shells. The morphology of the ZnO@AC composites was characterized by SEM (Fig. S4†) and TEM. Fig. 2a and b clearly show the multiple inner cores of ZnO NPs (the diameter of about 100 nm), outer shells of amorphous carbon (the thickness of about 90 nm), and outstanding void spaces between the cores and the shells. The diameter of ZnO NPs in the ZnO@AC composites was about 10 nm smaller than the diameter of pure ZnO NPs without carbon capping (Fig. S3†). The lattice fringes are clearly observable with an interlayer spacing of about 0.28 nm (Fig. 2c), which corresponds to the (100) plane of the ZnO wurtzite structure (JCPDS no. 36-1451). An HAADF-STEM image (Fig. 2d) and the corresponding elemental mapping (Fig. 2e–h) were obtained to further check the elemental composition. The distribution of C and N confirmed that the surface of ZnO was successfully coated with N-doped carbon shells. O and Zn were homogeneously distributed in the inner zone of the ZnO@AC composites, which was in agreement with the TEM and HAADF-STEM images.

**Fig. 3:**
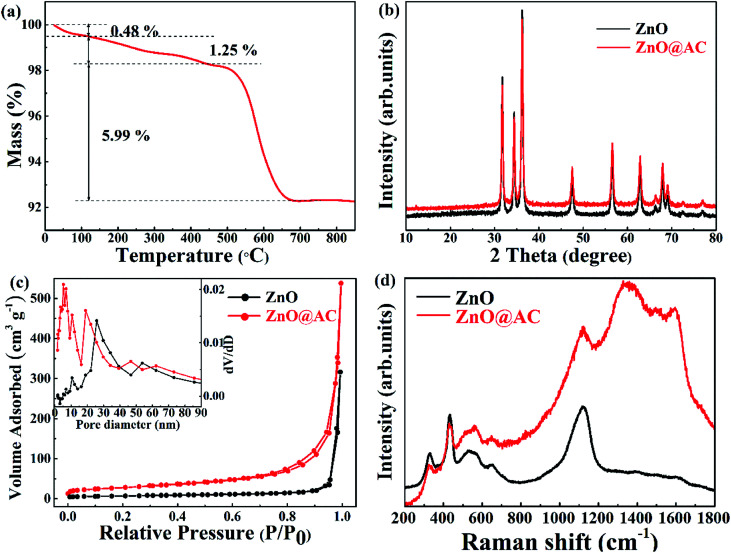
(a) TGA curve of ZnO@AC composites. (b) XRD data of pure ZnO NPs and ZnO@AC composites. (c) N_2_ adsorption/desorption isotherms of pure ZnO NPs and ZnO@AC composites. The inset figure shows the BJH pore size distributions of pure ZnO NPs and the ZnO@AC composites. (d) Raman spectra of pure ZnO NPs and the ZnO@AC composites.

Fig. 3a shows the TG results of the ZnO@AC composite in an air atmosphere. The sample showed three obvious weight loss stages. At the first stage, a slight weight loss (0.48 wt%) was observed below 120 °C, and it could be assigned to the loss of absorbed water. At the second stage, a minor weight loss of 1.25 wt% was observed over the temperature range from 120 °C to 450 °C, which was due to the loss of functional groups.^33,34^ At the third stage, a major weight loss of 5.99 wt% was observed over the temperature range from 450 °C to 700 °C, which was caused by the combustion of amorphous carbon. Almost no weight loss was observed as the temperature increased. The mass loss indicates the feasibility of the carbon loading on the composites. Correspondingly, the amount of residual ZnO was calculated to be approximately 92.28 wt% in the ZnO@AC composites.

The crystal phases of pure ZnO NPs and the ZnO@AC composites were confirmed by X-ray diffraction (Fig. 3b). All the diffraction peaks can be indexed to wurtzite ZnO (JCPDS no. 36-1451). For the ZnO@AC composites, no diffraction peak corresponding to the amorphous carbon shells was observed, implying the amorphous nature and the low loading content of the carbon shells. The diffraction peaks for the ZnO crystals in both samples were sharp and intense, indicating the highly crystalline nature of ZnO. No impurity peaks were observed, confirming the high purity of the samples. The size of the ZnO particles, as determined using the Scherrer equation, was approximately 98 nm, which was in good agreement with the TEM measurement.

The nitrogen adsorption–desorption isotherms and the pore-size distribution of the samples were observed using the Brunauer–Emmett–Teller (BET) method. Pure ZnO NPs exhibited fairly low porosity with a specific surface area of 23.445 m^2^ g^−1^ and a pore volume of 0.3918 m^3^ g^−1^. For the ZnO@AC composites, the specific surface area and pore volume were found to be 96.759 m^2^ g^−1^ and 0.5711 m^3^ g^−1^, respectively, which were much higher than those of pure ZnO NPs. As shown in Fig. 3c, both isotherms are typical IV isotherms, which suggests a dominant mesoporous structure.^30^ Pore volumes and pore size distributions (inset in Fig. 3c) were observed using the Barrett–Joyner–Halenda (BJH) method. In pure ZnO NPs, the large-size of pores (around 26 nm) was attributed to the spaces between the particles. In the case of the ZnO@AC composites, two kinds of pores could be observed. The large pores of around 20 nm were attributed to the spaces between the particles encapsulated in the AC shells, which were smaller than those of pure ZnO NPs. The tiny pores of around 6 nm were attributed to the developed porous structure of the AC shells, which could obtain numerous tiny channels for lithium ion diffusion during cycling.

Under ambient conditions, the zone center Raman active optical phonons of wurtzite ZnO (hexagonal phase) in the space group *P*6_3_*mc* are predicted by the group theory to be *Γ* = A_1_ + E_1_ + 2E_2_ (Raman active). The A_1_ branch originates from the ion vibrations along the *c*-axis, and the E_1_ branch is related to the ion vibrations perpendicular to the *c*-axis. The A_1_ and E_1_ branches are polar modes, and they split into longitudinal-optical (LO) and transverse-optical (TO) components with different frequencies. The two E_2_ branches are nonpolar phonons. The high-frequency E^high^_2_ mode is associated with the O motion, and the low-frequency E^low^_2_ mode is mainly associated with the Zn motion.^35,36^ The Raman spectra of pure ZnO NPs and the ZnO@AC composites are shown in Fig. 3d. The mode of A_1_(E^high^_2_ − E^low^_2_) at 331 cm^−1^ and the overtone of A_1_(E^high^_2_ − E^low^_2_) at 663 cm^−1^, A_1_(E^high^_2_) at 436 cm^−1^, A_1_(LO) at 541 cm^−1^ and A_1_(2LO) at 1098 cm^−1^ were found in both pure ZnO NPs and the ZnO@AC composites. These are the characteristic Raman signals of wurtzite ZnO and consistent with the results of the group theory analysis. Two strong peaks at around 1348 cm^−1^ and 1592 cm^−1^ were observed for the ZnO@AC composites and were assigned to the D and G bands of carbon, respectively.^25,29^ These two typical modes confirm the presence and partial graphitization of carbon. The ZnO@AC nanocomposite retained the Raman active modes of both ZnO and AC. Compared with the results for ZnO, very similar peak positions were observed for the ZnO@AC nanocomposites. No Raman peak shifts could be observed. However, the intensity of A_1_ and E^high^_2_ significantly decreased, which was ascribed to the weakening of the O–Zn bond.^28^

**Fig. 4:**
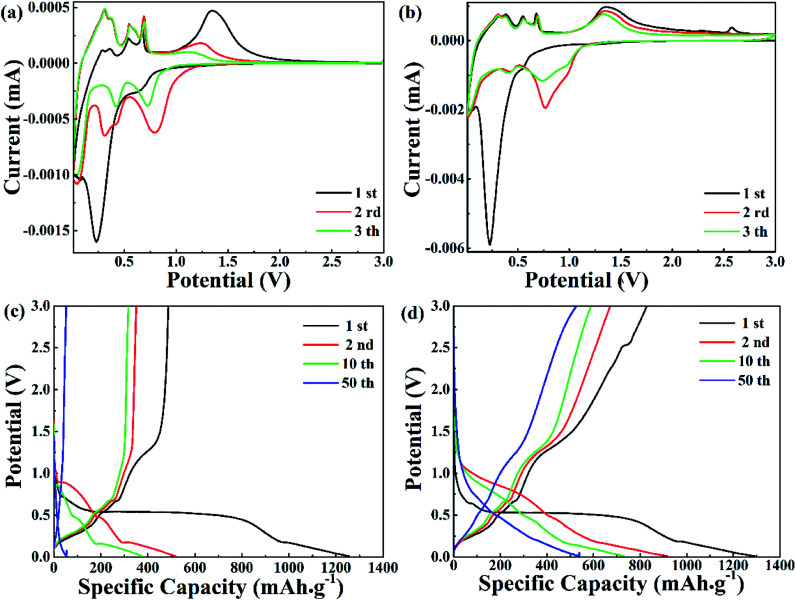
(a and b) Representative cyclic voltammetry (CV) curves within a voltage range of 0.01–3.0 V *vs.* Li/Li^+^ at a scan rate of 0.5 mV s^−1^. (c and d) Charge–discharge voltage profiles at a current density of 0.1 A g^−1^.

The electrochemical properties of pure ZnO NPs and the ZnO@AC composites were characterized using cyclic voltammetry (CV) and galvanostatic charge–discharge cycling. Fig. 4a and b show the CV plots of pure ZnO NPs and the ZnO@AC composites for the initial three cycles. Similar configurations of the CV curves were observed for pure ZnO NPs and the ZnO@AC composites, indicating that the amorphous carbon shells could barely change the reaction mechanism between ZnO and lithium. In the first cathodic scan, a pronounced peak at 0.23 V and a broad peak at around 0.69 V were observed for both pure ZnO NPs and the ZnO@AC composites. The former peak corresponded to the decomposition of the electrolyte, the reaction of the lithium–zinc alloy, and the growth of a solid electrolyte interphase (SEI) layer.^37,38^ The latter was related to the reduction of ZnO to Zn and the generation of amorphous Li_2_O. These cathodic peaks shifted to higher potentials upon cycling, which may be ascribed to the gradual stabilization of the electrode reactions. In the subsequent anodic scan, several peaks were observed between 0 and 0.7 V. As reported in the literature, these peaks are associated with the multistep delithiation process of the Zn–Li alloy through LiZn → Li_2_Zn_3_ → LiZn_2_ → Li_2_Zn_5_ → Zn and the decomposition of the SEI layer.^28^ Besides, a broad peak at around 1.37 V is related to the reformation of ZnO by the reversible redox reaction between Zn and Li_2_O. For the ZnO@AC composites, a new anodic peak at around 2.53 V was observed in the first cycle but disappeared in the following scans. This may be due to the irreversible redox reaction between amorphous carbon and Li_2_O. Even so, with an increase in the number of cycles, the CV curves of the ZnO@AC composites indicated better reproducibility and lower current density decay than those of pure ZnO NPs. The advantages of the ZnO@AC anode indicate good electrochemical performances.

In Fig. 4b and c, the voltage *versus* capacity profiles of pure ZnO NPs and the ZnO@AC composites for the 1st, 2nd, 10th and 50th cycles are observed at a current density of 0.1 A g^−1^ in the potential range of 0.01–3.0 V. Associated with the lithiation and delithiation processes, the plateaus observed in the discharge and charge voltage profiles are consistent with the anodic and cathodic peaks in the CV curves. Compared with pure ZnO NPs, the slow shrinkage of the charge/discharge curves of the ZnO@AC anode exhibited better capacity and stability upon cycling. The initial charge and discharge capacities of the ZnO@AC anode were found to be 1292 and 833 mA h g^−1^, respectively, corresponding to a coulombic efficiency (CE) of 64%. This CE of the ZnO@AC composite electrode was much higher than that of the pure ZnO NP anode (39%). The high capacity was achieved from the synergistic effect of the inner multiple nanoscale ZnO particles and the outer porous AC shell structure, which was in good agreement with the CV results. The protection of the amorphous carbon shell is the decisive factor to avoid detrimental reactions between ZnO NPs and the electrolyte, which enables the full utilization of active ZnO NPs wrapping in the AC shells.

**Fig. 5:**
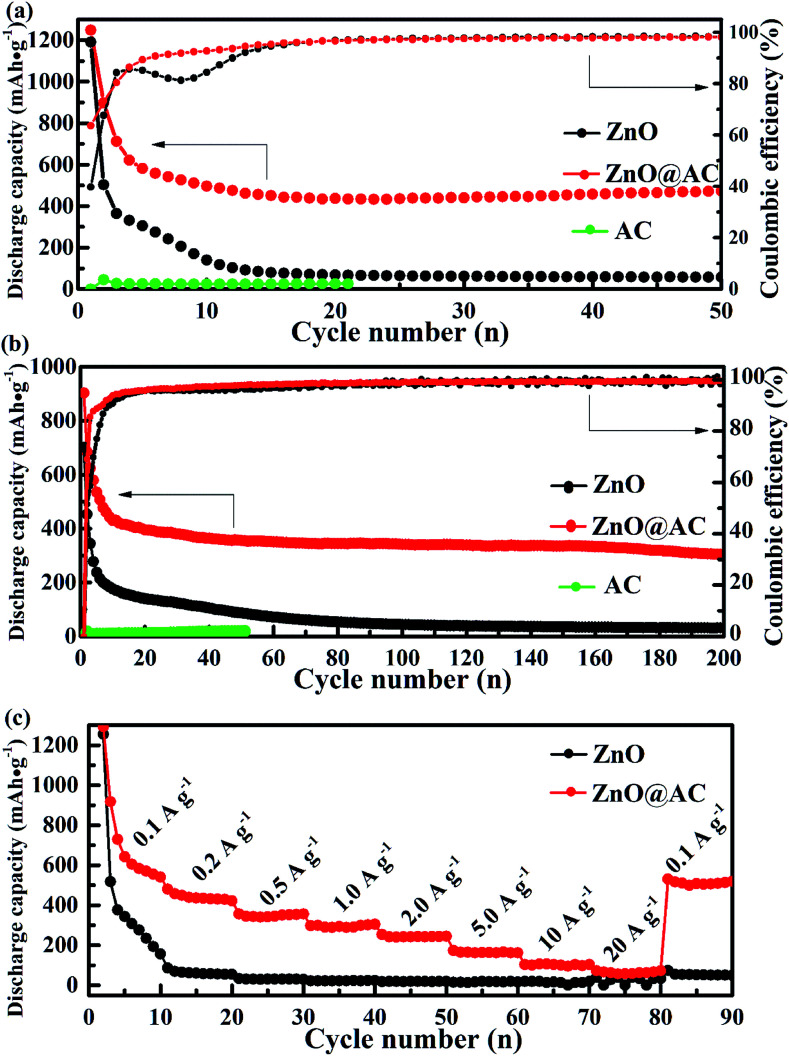
Electrochemical performances of (a) long cycle performance and CE at the current densities of 0.1 A g^−1^ and (b) 1 A g^−1^, and (c) the rate performance of pure ZnO NPs, the ZnO@AC composites and AC.

To verify the cycling performance of pure ZnO NP and ZnO@AC composite electrodes, long-term cycling tests and the corresponding CEs are shown in Fig. 5a and b. The ZnO@AC anode retained a capacity of 475 mA h g^−1^ (with a CE of 98.8%) after 50 cycles at the current density of 0.1 A g^−1^, which was nearly 9 times higher than that of the pure ZnO NP anode (∼57 mA h g^−1^). Even at a high current density of 1 A g^−1^, the ZnO@AC anode still delivered a capacity of 338 mA h g^−1^ (with a CE of 99.2%) after 200 cycles, which was 10 times higher than that of the pure ZnO NP anode (∼35 mA h g^−1^). Fig. 5c shows the rate performance of the pure ZnO NP and ZnO@AC anodes cycled at various current densities from 0.1 to 20 A g^−1^. As can be seen, the ZnO@AC anode delivers stable reversible capacities of 570, 436, 341, 289, 250, 160, 106, and 87 mA h g^−1^ at variable currents of 0.1, 0.2, 0.5, 1, 2, 5, 10, and 20 A g^−1^, respectively. When the current was reduced back to 0.1 A g^−1^, a capacity of 511 mA h g^−1^ was recorded for the ZnO@AC anode, which was almost 90% of the previous capacity obtained at the same rate. The ZnO@AC anode shows high capacity, excellent cycling stability, and competitive rate performance. These superior performances of the ZnO@AC composites can be attributed to three unique features. First, the ZnO@AC composites with a large specific surface area and numerous tiny pores can make full utilization of the active ZnO NPs to store lithium ions, thus providing high capacity. Second, the dispersion and morphology of the encapsulated ZnO NPs provide a confined diffusion distance and multidimensional lithium ion transport pathways to increase the rate capacity. Third, the void space between ZnO NPs and AC shells is an important factor to accommodate the large volume expansion during the lithiation–delithiation processes, balance the stress over the whole composite electrode and prevent aggregation over repeated cycling, which can improve the cycling stability and rate performance.

**Fig. 6:**
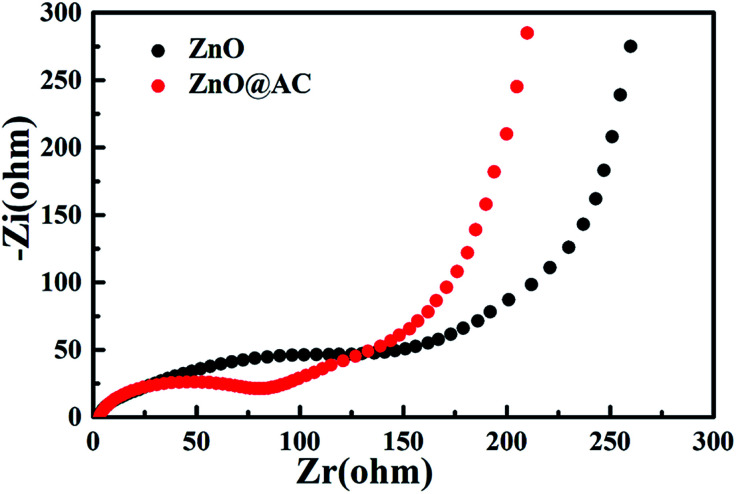
Electrochemical impedance spectra of pure ZnO NPs and the ZnO@AC composites.

Electrochemical impedance spectroscopy (EIS) of pure ZnO NPs and the ZnO@AC composites was performed using fresh cells. Fig. 6 presents the Nyquist plots of pure ZnO NPs and the ZnO@AC composites, which share similar configurations with a semicircle at high-to-medium frequencies and a sloping Warburg tail at low frequencies. The semicircle in the high frequency region is attributed to the charge transfer resistance (*R*_ct_) at the electrode–electrolyte interface, and the inclined line (the Warburg tail) in the low frequency region corresponds to diffusion impedance (*Z*_w_).^14,20^ As indicated in the figure, the *R*_ct_ and *Z*_w_ of the ZnO@AC composites are much lower than those of ZnO NPs, which demonstrate the above-mentioned structural merits of the ZnO@AC composites.

## 4. Conclusion

In summary, ZnO@AC composites were fabricated using a simple and moderate preparation method involving a dopamine polymerization and the subsequent annealing process of polydopamine carbonization together with precursor decomposition. The as-synthesized ZnO@AC composites delivered enhanced coulombic efficiency, high reversible capacity, improved rate capability, and long cycling lifespan due to the structural advantages, which originated from the unique multi-core–shell structure. All these results make the ZnO@AC composite a promising electrode material for advanced high-performance rechargeable batteries. Moreover, by taking advantage of this rational synthesis strategy and the unique multi-core–shell structure, our study sheds light on the design of high-performance electrodes for LIBs.

## Conflicts of interest

There are no conflicts to declare.

## Supplementary Material

RA-010-D0RA02497J-s001
