# Supplementary material for: Template-free synthesis and lithium-ion storage performance of multiple ZnO nanoparticles encapsulated in hollow amorphous carbon shells
Source: RSC Adv. 2020 Jun 15;10(38):22848–55. doi: 10.1039/d0ra02497j (PMC9054705; doi:10.1039/d0ra02497j)
Supplement: RA-010-D0RA02497J-s001 [file RA-010-D0RA02497J-s001.pdf]

Electronic Supplementary Material (ESI) for RSC Advances.
This journal is © The Royal Society of Chemistry 2020

Supporting Information

Template Free Synthesis and Lithium Ion Storage Performance of Multiple ZnO Nanoparticles @ Hollow Amorphous Carbon Shell

Yunxia Jin [1,2, 3*], Shimin Wang[2,3], Jia Li[1], Sheng Qu[1], Liufang Yang[1], Junming Guo[2,3*]

1.School of Electrical and Information Tehnology, Yunnan Minzu University, Kunming 650500, China.

2.School of Chemistry and Environment, Yunnan Minzu University, Kunming 650500, China.

3.National and Local Joint Engineering Research Center for Green Preparation Technology of Biobased Materials, Yunnan Minzu University, Kunming 650500, China.

* Corresponding authors.

Email: jyx1601@aliyun.com; guojunmingkm@163.com

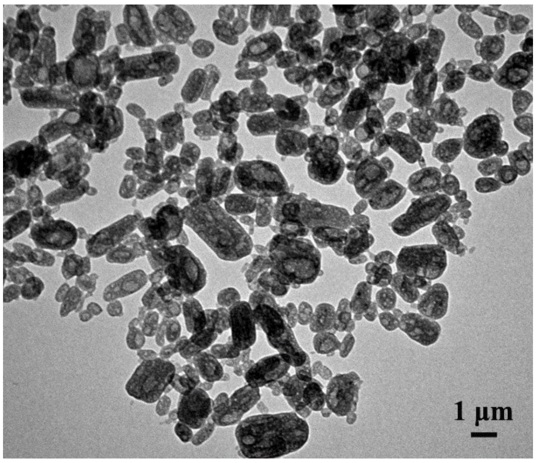

Fig. S1 TEM image of zinc hydroxide carbonate nanoparticles.

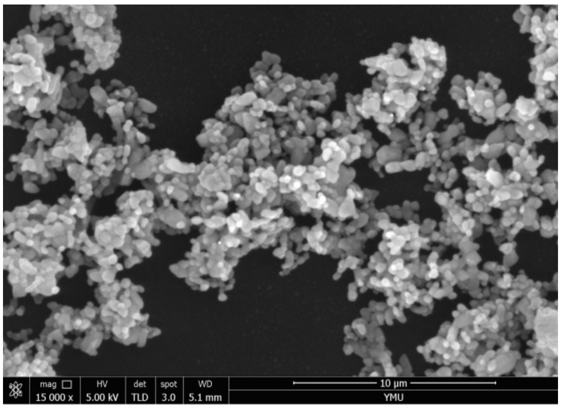

Fig. S2 SEM image of zinc hydroxide carbonate nanoparticles.

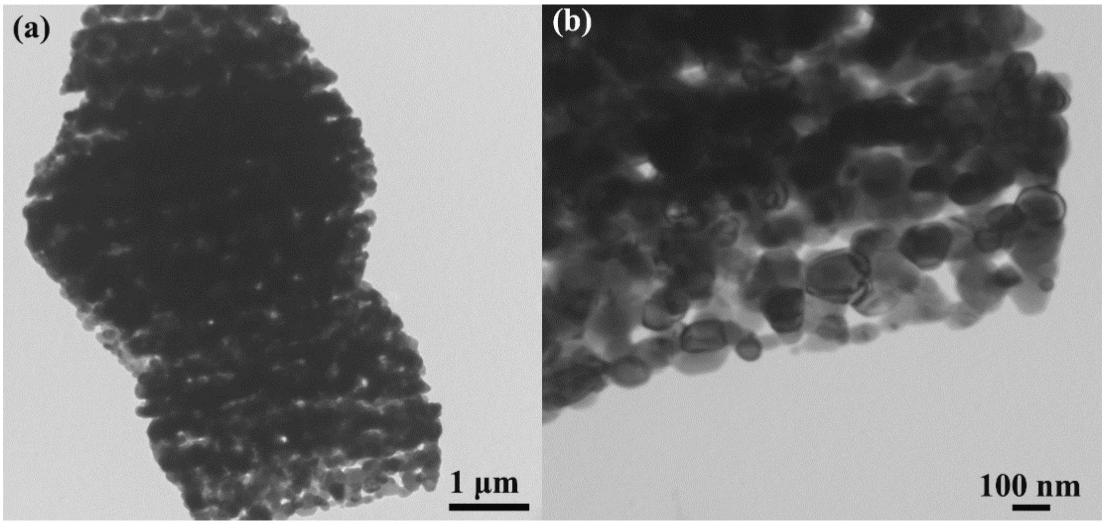

Fig. S3 TEM (a) and enlarged TEM image (b) of pure ZnO nanoparticles.

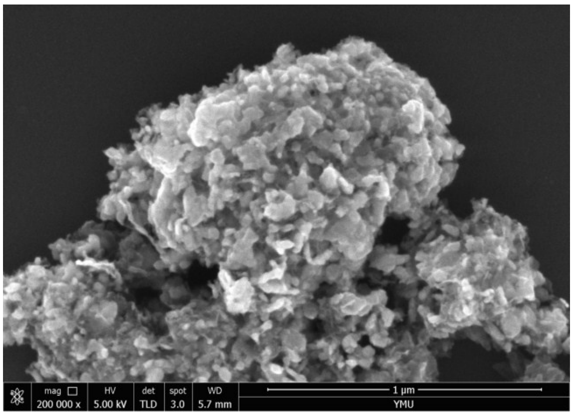

Fig. S4 SEM image of ZnO@AC.
